# Internet Searches for Lorazepam Following the Release of *The White Lotus*

**DOI:** 10.1001/jamahealthforum.2025.4931

**Published:** 2025-11-14

**Authors:** Kevin H. Yang, Nora Satybaldiyeva, Wayne Kepner, Joseph Friedman, Eric C. Leas

**Affiliations:** 1 Department of Psychiatry, University of California San Diego School of Medicine, La Jolla; 2 Stanford Prevention Research Center, Stanford University School of Medicine, Palo Alto, California; 3 Department of Psychiatry and Behavioral Sciences, Stanford University School of Medicine, Palo Alto, California; 4 Herbert Wertheim School of Public Health and Human Longevity Science, University of California, San Diego, La Jolla

## Abstract

This cross-sectional study examines whether there was an uptick in online searches about lorazepam after the release of the third season of *The White Lotus*.

## Introduction

On February 16, 2025, *HBO Max* released the third season of *The White Lotus*, which featured a central storyline in which a character repeatedly used lorazepam for anxiety, with frequent on-screen references to this benzodiazepine. Given that shows on streaming platforms can contribute to public interest and health behaviors^1,2^ and lorazepam carries misuse risk,^3^ we evaluated whether *The White Lotus* season 3 release was associated with increased public interest in lorazepam and compared trends with other commonly prescribed benzodiazepines.

## Methods

We obtained weekly US search engine (Google) data for *lorazepam*, *alprazolam*, and *clonazepam* from January 1, 2022, through June 6, 2025. We also examined searches that potentially indicated an attempt to acquire each benzodiazepine (eg, “where can I order [benzodiazepine] online?”) using a composite query string: *[benzodiazepine] online* + *[benzodiazepine] order* + *[benzodiazepine] buy* + *[benzodiazepine] get* + *[benzodiazepine] shop*. Search rates were measured as a fraction of total searches and expressed per 10 million. Trends were obtained from the Google API Client in Python.

Our approach was quasiexperimental, comparing observed search rates following the release of the third season of *The White Lotus* on February 16, 2025, until the time of analyses (June 6, 2025) with expected rates for this period. Expected search rates were calculated using an autoregressive integrated moving average model, with parameters selected using the Hyndman and Khandakar algorithm.^4^ Percentage increases were calculated as the ratio of observed to expected rates with bootstrapped 95% CIs. Absolute search volumes were estimated by multiplying observed search rates by total search estimates (eMethods in Supplement 1).

Analyses were performed using R, version 4.3.1 (R Foundation). The UC San Diego institutional review board considered the analyses exempt human participants research. This study followed the Strengthening the Reporting of Observational Studies in Epidemiology (STROBE) reporting guideline.

## Results

**Figure 1.**
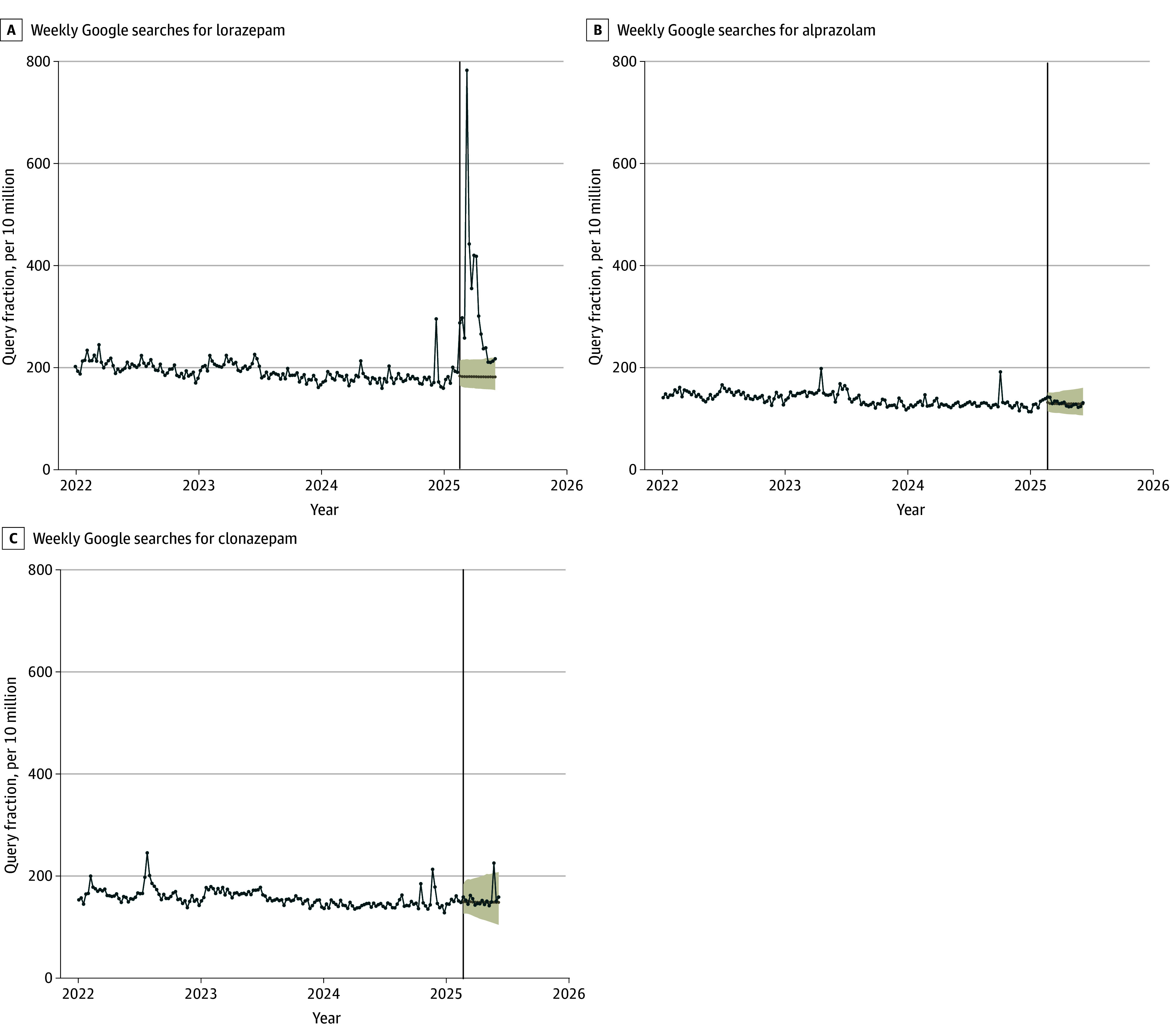
Weekly Online Searches for Lorazepam and Other Benzodiazepines Following the Release of the Third Season of *The White Lotus* Weekly online search volumes for select benzodiazepine medications in the US from January 1, 2022, through June 6, 2025. The vertical line indicates the release of third season of *The White Lotus* on February 16, 2025. Shaded areas represent 95% prediction intervals around the expected search volumes had the show not been released, as predicted by the ARIMA forecasting model. Search rates are expressed per 10 million total online searches. Data obtained from Google Trends.

Weekly search rates for lorazepam remained stable from January 2022 through the first week of February 2025, then increased following the release of the third season of *The White Lotus* (February 16, 2025) and remained elevated for 12 additional weeks (through the week of May 4, 2025) (Figure 1). During this 12-week period, lorazepam queries were cumulatively 98.6% (95% CI, 61.7%-149.1%) higher than expected, representing approximately 1.6 million additional searches. During the same time frame, queries for alprazolam and clonazepam remained at expected levels, at 1.4% (95% CI, −1.0% to 3.8%) and 0.7% (95% CI, −1.5% to 3.1%) of expected volumes, respectively.

**Figure 2.**
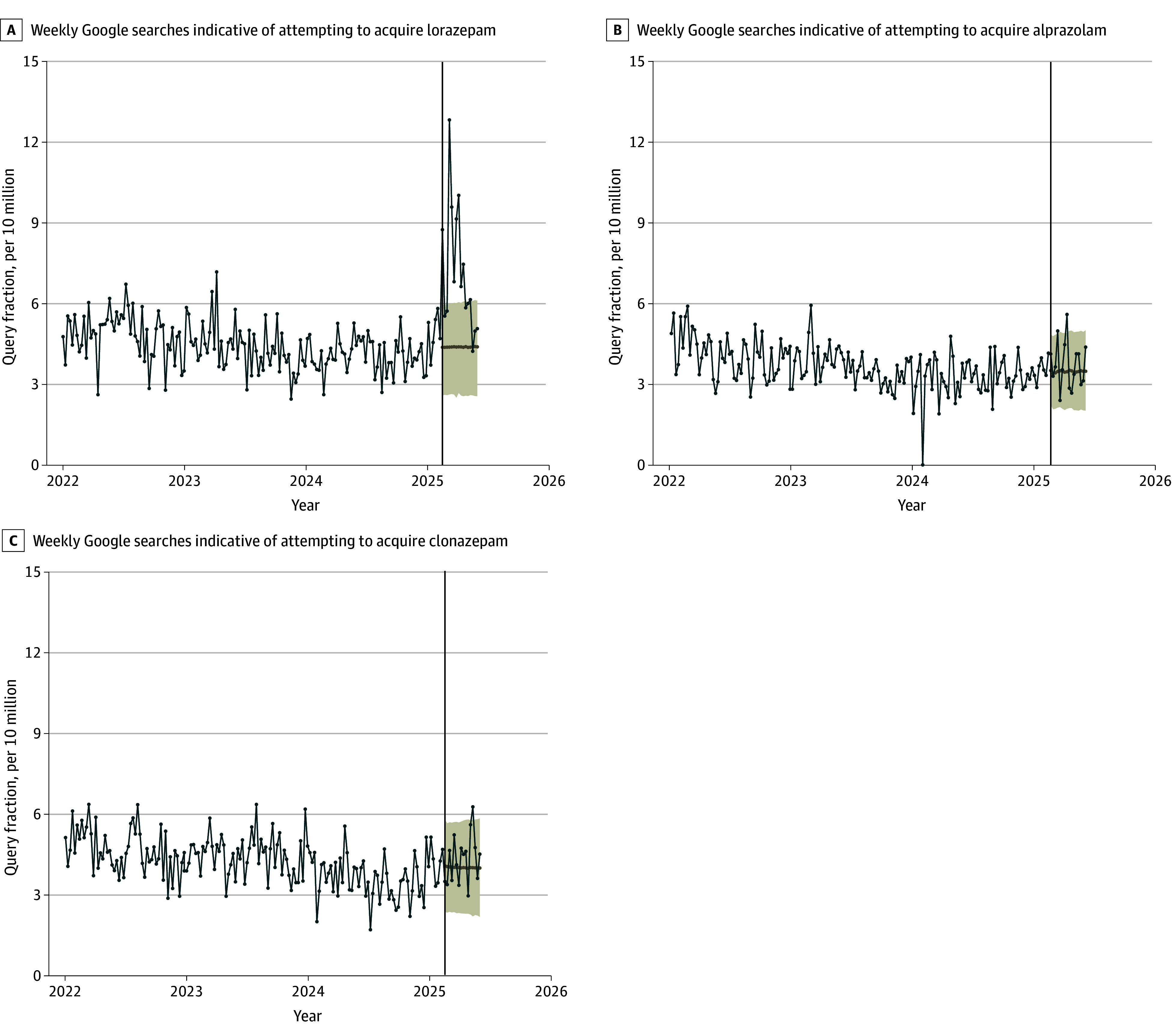
Weekly Online Searches for Benzodiazepine Acquisition Following the Release of the Third Season of *The White Lotus* Weekly online search volumes for acquisition-related queries for lorazepam, alprazolam, and clonazepam in the US from January 1, 2022, through June 6, 2025. Acquisition searches included the following composite query string: *[benzodiazepine] online* + *[benzodiazepine] order* + *[benzodiazepine] buy* + *[benzodiazepine] get* + *[benzodiazepine] shop*. The vertical line indicates the release of the third season of *The White Lotu*s on February 16, 2025. Shaded areas represent 95% prediction intervals around the expected search volumes had the show not been released, as predicted by the ARIMA forecasting model. Search rates are expressed per 10 million total online searches. Data obtained from Google Trends.

Searches that indicated an attempt to acquire lorazepam also increased and were cumulatively 63.6% (95% CI, 38.1%-90.2%) higher than expected for the 12-week period, representing approximately 30 000 additional searches. However, searches for acquiring alprazolam and clonazepam remained at expected levels during the same period, at 7.5% (95% CI, −4.3% to 20.4%) and 7.9% (95% CI, −3.2% to 18.8%) of expected volumes, respectively (Figure 2).

## Discussion

This cross-sectional study suggests that the release of the third season of *The White Lotus* was associated with increased public interest in lorazepam, generating 1.6 million excess searches. This increase, which also included queries associated with acquiring lorazepam online, was not observed for other benzodiazepines and aligned with prior research on how the media is associated with information-seeking and behavior.

Although a limitation of this study is that the findings may reflect information seeking rather than actual medication use, they nonetheless carry clinical implications. The rise in benzodiazepine prescribing^5^ and illegitimate online pharmacies selling benzodiazepines without prescriptions^6^ raises concerns about potential misuse. Clinicians may also encounter patients whose interest in these medications has been shaped by media exposure rather than clinical need. Moreover, the show did not portray the risks of combining benzodiazepines with alcohol (eg, respiratory depression) or abrupt cessation (eg, panic attacks, agitation, and seizures).^3^

Establishing best practices for depicting prescription medications with misuse potential may minimize unintended public health consequences. Additionally, optimizing health information panels (info boxes) to provide accurate, evidence-based information and support resources could help meet entertainment-driven curiosity.

## Notes

Supplement 1. eMethods. Expanded Discussion of Methods eReferences.

Supplement 2. Data sharing statement
