# Supplementary material for: Internet Searches for Lorazepam Following the Release of The White Lotus
Source: JAMA Health Forum. 2025 Nov 14;6(11):e254931. doi: 10.1001/jamahealthforum.2025.4931 (PMC12619094; doi:10.1001/jamahealthforum.2025.4931)
Supplement: Supplement 1. — eMethods. Expanded Discussion of Methods eReferences. [file jamahealthforum-e254931-s001.pdf]

Supplemental Online Content

Yang KH, Satybaldiyeva N, Kepner W, Friedman J, Leas EC. Internet searches for lorazepam following the release of *The White Lotus*. *JAMA Health Forum*. 2025;6(11):e254931.
doi:10.1001/jamahealthforum.2025.4931

eMethods. Expanded Discussion of Methods
eReferences.

This supplemental material has been provided by the authors to give readers additional information about their work.

© 2025 Yang KH et al. *JAMA Health Forum*.

eMethods. Expanded Discussion of Methods

Google Trends does not explicitly provide the total number of searches. We calculated an approximation for the period of interest (January 1, 2022 - June 6, 2025) using the following process. First, we obtained the search total originating from US desktop computers for the 3.5 years from January 1, 2022 through June 6, 2025 from comscore.com.[1] Second, we adjusted this number by the fraction of searches originating from desktop computers in the United State (50.2%) estimated by the firm MobiLoud using their proprietary database.[2] Third, we relied on the Google Search API to obtain the query fraction (QF) for searches matching those in our study, reflected as the number of searches matching per 10 million Google searches. Finally, we calculated an estimate for the total number of searches during this period using the following equation: [QFpo – E(QFpo)] • CSt • AF; where QFp is the query fraction (in this case expressed per 1 search) for the period of interest p, E(QFpo) is the expected QFp for the period based on historical forecasts from the ARIMA model. CSt is the comscore estimate of total monthly desktop search total available at the time of analysis t, and AF is the adjustment factor for the estimated proportion of Google searches that originated from desktop computers.

© 2025 Yang KH et al. *JAMA Health Forum*.

eReferences

1. Rankings. Comscore, Inc. Accessed July 2, 2025. https://www.comscore.com/Insights/Rankings

2. MobiLoud - Convert Your Website to Native Mobile Apps. Accessed July 2, 2025. https://www.mobiloud.com

© 2025 Yang KH et al. *JAMA Health Forum*.
