# Supplementary material for: Internet Searches for Lorazepam Following the Release of The White Lotus
Source: JAMA Health Forum. 2025 Nov 14;6(11):e254931. doi: 10.1001/jamahealthforum.2025.4931 (PMC12619094; doi:10.1001/jamahealthforum.2025.4931)
Supplement: Supplement 2. — Data sharing statement [file jamahealthforum-e254931-s002.pdf]

Data Sharing Statement

Yang. Internet Searches for Lorazepam Following the Release of The White Lotus. *JAMA Health Forum*. Published November 14, 2025. doi:10.1001/jamahealthforum.2025.4931

Data
Data available: No
